# A lignocellulose-degrading synthetic microbial community alters physicochemical properties, microbial community structure, and functional gene potential during green waste-cattle manure co-vermicomposting

**DOI:** 10.3389/fmicb.2026.1927926

**Published:** 2026-09-09

**Authors:** Linlin Cai, Shuang Pang, Dan Hao, Xiangyang Sun, Ximei Zhang, Muhammad Atif Muneer, Honghui Wu

**Affiliations:** 1 State Key Laboratory of Efficient Utilization of Arid and Semi-arid Arable Land in Northern China, Institute of Agricultural Resources and Regional Planning, Chinese Academy of Agricultural Sciences, Beijing, China; 2 Key Laboratory of Arable Land Quality Monitoring and Evaluation, Ministry of Agriculture and Rural Affairs, Institute of Agricultural Resources and Regional Planning, Chinese Academy of Agricultural Sciences, Beijing, China; 3 Experimental Centre of Forestry in North China, Chinese Academy of Forestry, Beijing, China; 4 Key Laboratory for Silviculture and Conservation of Ministry of Education, College of Forestry, Beijing Forestry University, Beijing, China; 5 College of Resource and Environment, International Magnesium Institute, Fujian Agriculture and Forestry University, Fuzhou, China

**Keywords:** carbohydrate-active enzyme genes, co-vermicomposting, high-lignin green waste, nitrogen cycling genes, synthetic microbial communities

## Abstract

Co-vermicomposting high-lignin green waste with cattle manure can improve nutrient supply and microbial activity; however, this technology remains limited in terms of lignocellulose degradation efficiency and product quality improvement. This study evaluated the effects of 5% calcium superphosphate (SSP), 5% synthetic lignocellulose-degrading microbial communities (SynComs), and their combined addition on vermicompost quality, microbial communities, and the functional potential related to carbohydrate degradation and nitrogen cycling. The results showed that all treatments maintained a neutral to slightly alkaline pH, favoring the growth of microorganisms involved in carbon and nitrogen cycling. Compared with other treatments, 5% SynComs showed the strongest stimulatory effect on lignocellulose-degrading enzyme and urease activities, effectively reduced the C/N ratio, and increased the germination index, humic substance content, nitrate nitrogen content, and overall quality score. Microbial analysis showed that the amendment treatments enriched nitrogen cycling related taxa, including *Luteimonas* and *Pseudomonas*. SynComs further increased the abundances of carbohydrate esterases and glycosyltransferases, as well as the relative abundance of the nitrification gene *hao*, thereby enhancing the potential for lignocellulose degradation, degradation product transformation, and nitrification. In contrast, SSP alone or combined with SynComs showed limited improvement in the activities and functional potential of enzymes related to carbohydrate and nitrogen metabolism. Overall, lignocellulose-degrading SynComs effectively accelerated co-vermicomposting maturity, improved product quality, and promoted green waste resource utilization. Future studies should optimize aeration conditions to reduce local anaerobic microenvironment formation and denitrification derived N_2_O emissions.

## 1 Introduction

Rapid urbanization and the expansion of urban green spaces have resulted in a continuous increase in green waste generation, which accounts for approximately 15–18% of total volume of urban solid waste (Luo et al., 2026; Jin et al., 2025). Green waste mainly consists of plant residues, including pruning branches, fallen litter, and deadwood, and is characterized by high lignocellulose content, heterogeneous composition, and a relatively high initial C/N ratio. Improper management of green waste can not only occupy valuable urban land resources but also promote the proliferation of pests and pathogens, resulting in environmental pollution and ecological risks (Feng et al., 2024; Li et al., 2025). Composting has been widely recognized as an effective strategy for the sustainable utilization of green waste. However, highly lignified materials, such as tree branches, trunks, and roots, contain recalcitrant lignocellulosic structures that are resistant to microbial degradation, thereby prolonging the composting process, limiting organic matter stabilization, and reducing the nutrient quality of the final compost product (Hoang et al., 2022). Co-composting green waste with cattle manure, which contains abundant readily degradable organic matter, nitrogen, and active microorganisms, can improve substrate nutrient balance and stimulate microbial activity. Furthermore, the incorporation of earthworms can enhance substrate transformation and microbial interaction through feeding, fragmentation, and gut-mediated biochemical processes. Therefore, co-composting with earthworms is considered a promising approach for the valorization of highly lignified green waste (Jin et al., 2025; Xia et al., 2025; Ding et al., 2026). Nevertheless, the persistent presence of recalcitrant lignocellulose remains a major constraint on carbon mineralization and nutrient release, limiting further improvements in the maturity and quality of vermicompost derived from co-vermicomposting of green waste and cattle manure.

Although cattle manure supplies readily available nutrients and active microorganisms to vermicomposting systems and creates favorable conditions for earthworm growth and microbial enzymatic activity, its addition alone cannot overcome the structural recalcitrance of lignocellulose in highly lignified green waste. Such waste mainly consists of pruned branches, roots and other woody residues, in which cellulose and hemicellulose are embedded within a highly cross-linked lignin matrix. This complex structure limits enzyme accessibility and confer strong resistance to microbial degradation, resulting in slow organic carbon transformation, prolonged composting periods, and insufficient nutrient release (Gong et al., 2019; Boutasknit et al., 2024; Chen et al., 2025a). Moreover, the marked differences in the physicochemical properties of green waste and cattle manure mean that their co-vermicomposting is governed not only by lignocellulose-degrading capacity but also by pH, nutrient stoichiometry, and the coupling of microbial carbon and nitrogen metabolism (Wang et al., 2023; Jin et al., 2025). Consequently, the use of cattle manure alone as a co-vermicomposting substrate is generally insufficient to simultaneously promote recalcitrant substrate transformation, regulate microbial functions, and improve vermicompost quality (Dai and Zhang, 2025; Luo et al., 2025). Combining inorganic phosphate amendment with functional microbial inoculation may provide a complementary strategy for overcoming these limitations. Calcium superphosphate (SSP) can regulate compost pH and nutrient availability, reduce nitrogen losses through volatilization, and influence microbial processes associated with organic matter degradation and carbon and nitrogen cycling (Wu et al., 2017; Zhao et al., 2023; Wang H. et al., 2025). Meanwhile, lignocellulose-degrading synthetic microbial communities (SynComs), constructed on the basis of interspecific metabolic complementarity can integrate cellulose, hemicellulose, and lignin degradation functions. Compared with single-strain inoculants, SynComs may therefore maintain more stable enzymatic activity and functional cooperation under complex and dynamically changing composting conditions (Zhang et al., 2024; Gad et al., 2024; Liu et al., 2025). Previous studies have separately demonstrated that SSP can enhance nutrient retention and compost maturation, whereas lignocellulose-degrading microbial consortia can accelerate organic matter transformation and alter microbial functions related to carbon and nitrogen metabolism (Zhao et al., 2023; Chen et al., 2025b; Yang et al., 2024). However, the combined effects of SSP and lignocellulose-degrading SynComs have not been systematically investigated in the co-vermicomposting of highly lignified green waste and cattle manure. In particular, it remains unclear whether and how these two amendments interact to regulate microbial community succession, lignocellulose degradation, and carbon and nitrogen metabolic potential, and thereby improve vermicompost maturity and quality.

SSP and lignocellulose-degrading SynComs may jointly regulate carbon and nitrogen transformation during the co-vermicomposting of green waste and cattle manure through complementary effects on the composting environment and microbial functions. SSP can modify substrate pH and phosphorus availability, enhance nitrogen retention, and reduce ammonia volatilization, thereby creating more favorable chemical conditions for microbial growth and organic matter transformation (Wu et al., 2017; Zhao et al., 2023; Wang et al., 2026). In parallel, lignocellulose-degrading SynComs can exploit metabolic complementarity among their functional members to enhance the activities of enzymes involved in cellulose, hemicellulose, and lignin degradation. This may accelerate the conversion of structurally complex organic carbon into readily available low-molecular-weight substrates and subsequently alter the ecological niches and functional potential of microorganisms involved in carbon metabolism, nitrogen mineralization, nitrification, and denitrification (Yao et al., 2024; Wang et al., 2023; Hoang et al., 2022). Earthworm feeding, gut-mediated transformation, and burrowing can further promote substrate fragmentation, microbial dispersal, and substrate and microbe contact, thereby strengthening the coupling between SSP mediated physicochemical regulation and SynCom mediated functional enhancement within the earthworm and microbe system (Gong et al., 2017; Xia et al., 2025). However, previous studies have largely examined inorganic phosphate amendment and functional microbial inoculation as separate strategies. It therefore remains unclear whether these two amendments interact in their effects on lignocellulose degradation, nitrogen retention, and humification, and whether such interactions are associated with changes in key microbial taxa and their carbon- and nitrogen-related metabolic functions. Moreover, the microbial groups and functional genes that drive these responses have not yet been clearly identified. Accordingly, an integrated assessment of physicochemical properties, enzyme activities, microbial community succession, and carbon and nitrogen cycling functions is required to elucidate the mechanisms by which SSP and lignocellulose-degrading SynComs regulate this co-vermicomposting system.

Based on the distinct roles of SSP in regulating physicochemical conditions and nutrient availability and of lignocellulose-degrading SynComs in promoting microbial functional activity, we hypothesized that SSP and SynComs would exert individual and interactive effects on carbon and nitrogen transformation during co-vemicomposting. However, whether SSP would enhance, weaken, or otherwise modify the functional effects of SynCom inoculation under complex vermicomposting conditions remained unclear. To address this question, SSP and lignocellulose-degrading SynComs were applied individually and in combination to evaluate their main and interactive effects on physicochemical properties, lignocellulose degradation, enzyme activities, nutrient transformation, maturity, microbial community structure, and carbon- and nitrogen-cycling functional potential. This study aimed to clarify how SSP modifies the effects of SynComs during co-vermicomposting and to identify the amendment strategy that most effectively improves vermicompost quality.

## 2 Materials and methods

### 2.1 Experimental materials

The green waste used in this experiment was obtained from the composting plant of the National Botanical Garden, Beijing, China. The raw material consisted of softwood and hardwood pruning branches that had been crushed to approximately 1 cm, with a moisture content of 18.7%. Cattle manure was purchased from Shijiazhuang Xushi Agricultural Technology Co., Ltd., and had a moisture content of 20.61%. The main physicochemical properties of the cattle manure and green waste are presented in Supplementary Table 1. Granular SSP was purchased from Zhengzhou Aipuda Agricultural Technology Co., Ltd. It had a particle diameter of 3–5 mm and an available phosphorus content of 12%. Before use, it was ground and passed through a 2-mm sieve. Its chemical properties were as follows: ammonium nitrogen content, 15.36 ± 0.20 mg/kg; pH, 3.28 ± 0.30; and electrical conductivity, 5.97 ± 0.03 mS/cm. Adult *Eisenia fetida*, with an average individual weight of approximately 0.45 g, were used for the vermicomposting experiments.

### 2.2 Isolation of lignocellulose-degrading microbial strains from compost

In our previous study on green waste composting (Yu et al., 2018), culturable lignocellulose-degrading strains were isolated from compost samples at the maturation stage using the serial dilution plating method. Briefly, 1.0 g of fresh compost sample was added to 10 mL of sterile phosphate-buffered saline (PBS; containing 0.15 M phosphate buffer and Tween 80, pH 7.0) and shaken at 28°C and 180 rpm for 60 min to prepare a 10^–1^ dilution. The suspension was then serially diluted to 10^–6^. Subsequently, 200 μL of each dilution was spread onto three screening media: PDA supplemented with 0.01% Azure B, PDA supplemented with 0.04% guaiacol, and PDA supplemented with 1% sodium carboxymethyl cellulose (CMC-Na) and 0.01% Congo red. After incubation, target strains were screened and purified based on the highest manganese peroxidase (MnP), lignin peroxidase (LiP), and laccase (Lac) activities. Genomic DNA was extracted from 0.1 g of each sample using the DNeasy Plant Mini Kit. For bacteria, the full-length 16S rRNA gene was amplified using the primer pair 27F/1492R, whereas for fungi, the ITS1 region was amplified using the primer pair ITS1F/ITS2. The obtained sequences were aligned against the NCBI BLAST database, and a phylogenetic tree was constructed using MEGA11 to determine species identity and phylogenetic relationships among the strains (Supplementary Figure S1).

Finally, three culturable strains with no antagonistic activity against the laboratory-preserved lignin-modifying fungus *Aspergillus nidulans* were selected (Yu et al., 2018): *Bacillus licheniformis* and *Bacillus subtilis*, which exhibited cellulase, LiP, or MnP activities, and *Aspergillus rugulosus*, which exhibited cellulase and Lac activities.

### 2.3 Construction of SynComs and establishment of the vermicomposting system

The SynCom was constructed using the three strains isolated in this study together with one laboratory-preserved strain, comprising two bacterial species (*Bacillus licheniformis* and *Bacillus subtilis*) and two fungal species (*Aspergillus nidulans* and *Aspergillus rugulosus*). A mixture design was employed to optimize the inoculation ratio of the four strains by maximizing the activities of lignin peroxidase (LiP), manganese peroxidase (MnP), and laccase (Lac). The solid-state fermentation substrate was prepared by mixing cattle manure and shredded green waste at a dry weight ratio of 3:7. The bacterial and fungal strains were cultured in LB and PDB media, respectively, until the late exponential growth phase. Bacterial cells were harvested by centrifugation and resuspended in sterile phosphate buffered saline (PBS), and the bacterial suspension was adjusted to a concentration of 1 × 10^8^ CFU/mL. *Aspergillus nidulans* and *Aspergillus rugulosus* were cultured for 5 days, after which conidia were collected by suspending them in sterile PBS containing 0.05% Tween-80. The conidial suspensions were passed through a 40 μm cell strainer to remove hyphal fragments. Spore concentrations were determined using a hemocytometer and adjusted to 1 × 10^8^ spores/mL before inoculation. The four standardized bacterial and fungal suspensions were then inoculated into the solid-state fermentation substrate at different volume ratios, with a total inoculum size of 5% (v/w) (Supplementary Table S2; Yu et al., 2018). Based on the enzyme activities measured after 35 days of incubation (Supplementary Table S3), the optimal SynCom composition was determined to be *Aspergillus nidulans*: *Bacillus licheniformis*: *Aspergillus rugulosus*: *Bacillus subtilis* = 1:2:2:1. This ratio was subsequently used for SynCom inoculation during vermicomposting. The same batch of standardized inoculum stock was used for all replicates at each inoculation event.

The vermicomposting experiment was conducted in 14.90 L (length × width × height, 35 cm × 20 cm × 21.29 cm) plastic reactors using a randomized block design with three replicates per treatment. Five treatments were established: (1) T1 (control), 1,000 g of green waste; (2) T2, 1,000 g of green waste supplemented with 358.95 g of cattle manure to adjust the initial C/N ratio to 25: 1; (3) T3, T2 supplemented with calcium superphosphate at 5% (w/w, dry weight basis), equivalent to a basal application rate of 70 g/m^2^; (4) T4, T2 inoculated with SynCom at 5% (v/w, based on the dry weight of the substrate) to achieve an initial microbial density of approximately 5.0 × 10^6^ microbial propagules g^–1^ dry substrate, comprising 2.5 × 10^6^ CFU g^–1^ dry substrate of bacterial cells and 2.5 × 10^6^ spores g^–1^ dry substrate of fungal spores; and (5) T5, T2 supplemented with both calcium superphosphate at 5% (w/w, dry weight basis) and SynCom at 5% (v/w, based on the dry weight of the substrate). All substrate components were calculated on a dry weight basis, and the initial moisture content was adjusted to 65% using tap water. The composting process consisted of a 15 days pre-composting stage followed by a 29 days vermicomposting stage. Calcium superphosphate and/or SynCom were applied on day 10 of pre-composting. Calcium superphosphate was added only once, whereas SynCom was re-inoculated every 7 days throughout the experiment. After sampling at the end of the pre-composting stage (day 15), 30 adults *Eisenia fetida* individuals were introduced into each reactor. To prevent earthworm escape, the reactors were covered with two layers of sterile gauze secured with rubber bands and incubated in the dark in a temperature-controlled chamber (RXZ-500A-LED, Ningbo Jiangnan Instruments, China) at 27 ± 0.5°C. During pre-composting and vermicomposting, substrate moisture content was measured every 7 days using an MS-70 moisture analyzer (A&D Co., Ltd., Tokyo, Japan) and maintained at 65 ± 5% by adding tap water as needed. During the final sampling, living *Eisenia fetida* individuals were still observed in all treatments, indicating that the experimental conditions supported earthworm persistence during the vermicomposting period. However, earthworm survival rate, biomass change, and avoidance behavior were not quantitatively evaluated in this study.

### 2.4 Sampling and physicochemical analysis

Composite samples were collected on days 1, 8, 15, 22, 29, 36, and 44. The composting materials were manually turned every 2 days to maintain aeration, and all turning and sampling tools were sterilized between treatments. Each composite sample consisted of approximately 20 g of subsamples collected from five positions within each reactor: the center, top, bottom, and both lateral sides of the middle layer. The samples were divided into three portions: one portion was air-dried for physicochemical analysis, another was stored at -4°C for enzyme activity assays, and the remaining portion was immediately stored at -80°C for microbial molecular ecological analysis. Because the physicochemical properties of the composting materials changed rapidly during the early stage, additional composite samples were collected on days 0 and 11 for microbial sequencing.

The pH and electrical conductivity (EC) were measured in aqueous extracts prepared at a sample: water ratio of 1:10 (w/v, on a dry weight basis) using an MP521 pH/conductivity meter (Shanghai, China). Total organic carbon (TOC) and total nitrogen (TN) were determined according to the Chinese agricultural industry standard (NY/T 525-2021). The germination index (GI), using Chinese cabbage (*Brassica rapa* L., Chinese group) seeds as indicator plants, was determined according to Gong et al. (2023) to evaluate phytotoxicity. Hemicellulose, cellulose, and lignin contents were determined using the Van Soest sequential fiber fractionation method with a Fibretherm FT12 automated fiber analyzer (C. Gerhardt, Germany) (Chen et al., 2025b). Total humic substances (HS) were extracted using a mixture of 0.1 M Na_4_P_2_O_7_⋅10H_2_O and 0.1 M NaOH, followed by acidification with 6 M HCl to separate humic acid (HA) and fulvic acid (FA) (Stevenson, 1994). The carbon content of each fraction was measured using a Shimadzu TOC-Vwp analyzer (Tokyo, Japan). After extraction with 2 M KCl at a ratio of 10:1 (v/v), NH_4_^+^-N and NO_3_^–^-N contents were determined using a continuous flow analyzer. Available potassium (AK) was measured using a 425-flame photometer (Shanghai, China). Cation exchange capacity (CEC) was determined using the sodium acetate saturation ammonium acetate exchange method (Bao, 2008). Microbial respiration-derived CO_2_ emissions were measured using the static incubation method (Jenkinson and Powlson, 1976). The activities of cellulase, urease, LiP, MnP, and Lac were determined. Fresh samples stored at -4°C were used for enzyme extraction. Briefly, 5.0 g of sample was extracted with 25 mL of 50 mM sodium acetate buffer (pH 5.0) at 4°C for 1 h with shaking, followed by centrifugation at 10,000 × g for 15 min. The collected supernatant was used as crude enzyme extract. Cellulase and urease activities were determined according to previous methods with modifications. LiP activity was measured in a reaction mixture containing crude enzyme extract, 125 mM sodium tartrate buffer and 0.1 mM H_2_O_2_, and the decrease in Azure B absorbance was monitored at 651 nm. MnP activity was determined using crude enzyme extract, 100 mM sodium malonate buffer (pH 4.5), and 10 mM MnSO_4_, with oxidation monitored at 270 nm. Laccase activity was determined using crude enzyme extract, 100 mM sodium malonate buffer (pH 4.5), and 6 mM ABTS, with absorbance measured at 420 nm. All reactions were performed at 25°C for 5 min. One unit (U) of enzyme activity was defined as the amount of enzyme causing a change of 0.001 absorbance units per minute under assay conditions, and enzyme activities were expressed as U g^–1^ fresh weight.

### 2.5 Metagenomic DNA extraction, sequencing, and bioinformatic analysis

Total genomic DNA was extracted from frozen samples collected at the initial, intermediate, and final stages of pre-composting and at the end of vermicomposting using the E.Z.N.A^®^ soil DNA kit (Omega Bio-tek, Norcross, GA, United States). Three biological replicates were analyzed for each treatment at each sampling stage, with 0.1 g of wet material used per extraction. DNA quality and concentration were assessed by 1% agarose gel electrophoresis and a NanoDrop 2000 spectrophotometer (Thermo Fisher Scientific, United States), respectively. Qualified DNA was used for paired-end library construction and metagenomic sequencing on the illumina NovaSeq platform by Shanghai Majorbio Bio-pharm Technology Co., Ltd.

Raw reads were quality filtered using fastp v0.20.0 and assembled with MEGAHIT v.1.1.2. Open reading frames were predicted using Prodigal v2.6.3, and a non-redundant gene catalog was constructed by clustering predicted sequences at 95% identity with CD-HIT v4.6.1. Gene abundance was estimated by mapping high-quality reads to the non-redundant gene catalog using Salmon v0.14.1. Gene sequences were aligned against the NCBI NR database using DIAMOND for taxonomic annotation. Functional annotation was performed against the KEGG and CAZy databases, with nitrogen cycling-related genes identified according to KEGG Orthology (KO) entries and carbohydrate-active enzymes annotated using dbCAN2 at an E-value threshold of < 1 × 10^–5^ (Liu et al., 2025). Sequencing and the principal bioinformatic analyses were completed on the Majorbio Cloud Platform.

### 2.6 Statistical analysis

Statistical analyses were performed in R 4.5.1 using appropriate statistical packages. The effect of cattle manure addition was evaluated by comparing T1 and T2, whereas T2-T5 were analyzed as a 2 × 2 factorial design to assess the main and interactive effects of SSP addition and SynCom inoculation. For variables measured repeatedly during composting, repeated-measures ANOVA was performed with composting time and the corresponding treatment factors and their interactions included in the model. Significant interactions involving composting time were further examined by comparisons at individual sampling times. Final product quality among T2–T5 on day 44 was assessed by one-way ANOVA followed by appropriate *post hoc* multiple comparisons. Statistical significance was set at *P* < 0.05, with *P* < 0.01 and *P* < 0.001 indicating increasing levels of significance.

## 3 Results and discussion

### 3.1 Effect of SSP and lignocellulose-degrading SynComs on lignocellulose degradation and microbial enzyme activity

Lignocellulose degradation is a key process driving the maturation of green waste vermicompost (Yao et al., 2024). Planned T1-T2 contrast analysis showed that cattle manure addition significantly enhanced lignocellulose degradation compared with green waste alone (Supplementary Table S4). Among the composite-substrate treatments (T2–T5), the combined SSP and SynCom treatment (T5) achieved the highest final degradation rates of lignin, cellulose, and hemicellulose (Supplementary Table S4), indicating a synergistic contribution of SSP and SynComs to lignocellulose decomposition.

**FIGURE 1:**
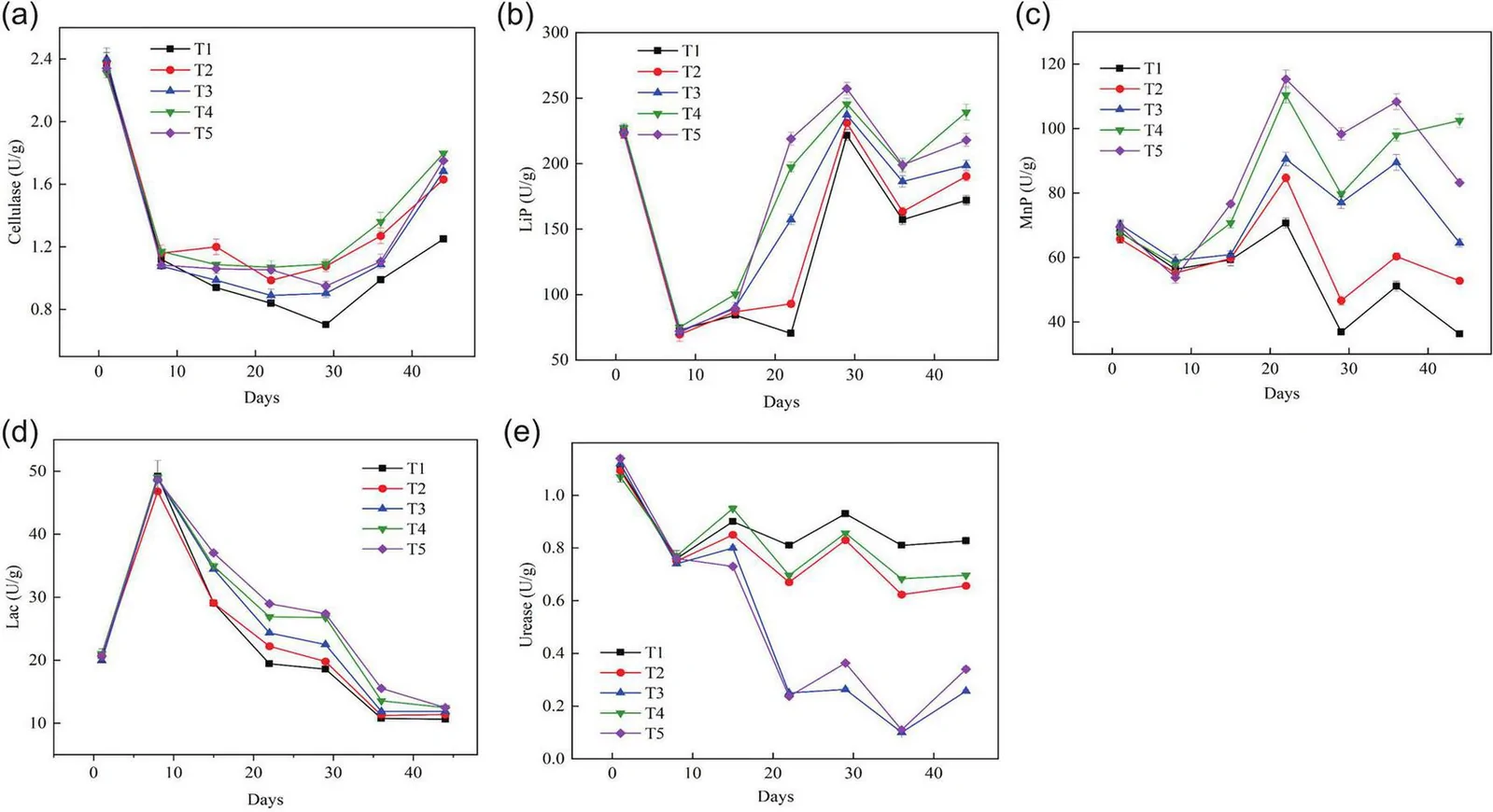
Effects of calcium superphosphate (SSP) and/or synthetic microbial community (SynComs) inoculation on hydrolytic enzyme activities during vermicomposting. **(a)** the activities of cellulase; **(b)** the activities of lignin peroxidase; **(c)** the activities of manganese peroxidase; **(d)** the activities of laccase; **(e)** the activities of urease. LiP, lignin peroxidase; MnP, manganese peroxidase; Lac, laccase. T1–T5 are described in section 2.3.

Changes in lignocellulose degradation were accompanied by marked shifts in enzyme activities. Cattle manure addition significantly increased cellulase activity by 27.66 and 30.40% on days 15 and 44, respectively. On day 44, cellulase activities in the SSP, SynCom, and combined treatments were 3.27, 10.22, and 7.36% higher than that in T2, respectively, with T4 showing the highest activity (1.80 U/g; Figure 1a). In contrast, SSP-containing treatments markedly reduced urease activity, with T3 and T5 showing 60.91 and 93.14% lower activities than T2 on day 44, respectively (*P* < 0.01; Figure 1e). Repeated-measures ANOVA further showed that SSP significantly reduced cellulase and urease activities, whereas SynComs enhanced late-stage cellulase activity and significantly promoted urease activity. These contrasting responses may reflect SSP-induced changes in pH and C/N ratio that constrain enzyme production by some microorganisms (Zhao et al., 2023), whereas SynComs enhance microbial functions associated with cellulose degradation and nitrogen cycling (Yang et al., 2024; Gong et al., 2023). The relatively high cellulase but low urease activity under the combined treatment may favor carbon decomposition while limiting nitrogen mineralization, thereby contributing to nitrogen retention (Ajibade et al., 2020).

SSP and SynComs also strongly affected lignin-degrading enzymes. Cattle manure addition increased LiP and MnP activities at both the middle and final stages, while SSP and/or SynCom addition further enhanced LiP, MnP, and Lac activities (Figures 1b–d). On day 44, T4 showed the highest LiP (239.20 U/g) and MnP (102.48 U/g) activities, whereas its Lac activity was comparable to that of T5. Repeated-measures ANOVA confirmed significant effects of SSP, SynComs, and their interaction on lignin-degrading enzyme activities (*P* < 0.05). LiP, MnP, and Lac act cooperatively in the degradation of lignin and aromatic compounds (Chen et al., 2025a), and their enhanced activities after earthworm addition may be related to earthworm-stimulated microbial activity and increased fungal growth (Wang Y. et al., 2025; Liu et al., 2025). However, earthworm growth and activity were not quantitatively evaluated in this study. Earthworm biomass, survival, and avoidance behavior may have varied among treatments and could have affected substrate aeration, microbial dispersal, and organic matter transformation, thereby potentially influencing lignin-degrading enzyme activities. The stronger response under SynCom treatment suggests that SynCom inoculation was associated with enhanced ligninolytic enzyme activities. Based on previous studies, this response may be related to functional complementarity among introduced and indigenous microorganisms and microbial interactions (Čaušević et al., 2024). SSP may further support fungal growth and enzyme secretion by supplying P, Ca, and S and regulating pH (Wang Y. et al., 2025). However, the lower ligninolytic enzyme activities under the combined treatment than under SynComs alone suggest that SSP modified the stimulatory effect of SynComs on lignin-degrading enzyme production.

### 3.2 Effect of SSP and lignocellulose-degrading SynComs on vermicompost maturity indicators

The initial pH values of all treatments ranged from 7.38 to 7.49 (Supplementary Figure S2a). After pre-composting, T1 and T2 showed higher pH values, likely due to stronger ammonification, whereas T3–T5 remained lower. By day 44, pH ranged from 7.53 to 7.99, with significantly lower values in the SSP-containing treatments (T3 and T5). Earthworm-mediated CaCO_3_ secretion and ammonia production may buffer acidification caused by SSP and organic acid accumulation, maintaining neutral to slightly alkaline conditions favorable for microbial activity (Ajibade et al., 2020; Versteegh et al., 2017; Du et al., 2025; Wang et al., 2023). SSP also shifted the final products closer to neutrality, potentially improving their suitability as seedling substrates (Dai and Zhang, 2025). EC increased markedly in T3 and T5 after pre-composting and remained relatively high in T3-T5 on day 44 (Supplementary Figure S2b), likely due to the release of soluble ions such as PO_4_^3–^ and Ca^2+^ from SSP. Nevertheless, final EC values (0.31–0.93 mS/cm) remained well below the 4 mS/cm threshold for moderately salt-sensitive plants (Wang Y. et al., 2025). Combined vermicomposting of green waste and cattle manure significantly reduced the C/N ratio, with final values of 16.14–18.67 in T3–T5, below the commonly used maturity threshold of 20 (Supplementary Figure S2c; Gong et al., 2023). This suggests that cattle manure improved substrate nutrient balance and promoted organic matter degradation and maturation. Product maturity was further supported by the germination index (GI), which exceeded 80% in all final products, indicating low phytotoxicity (Zucconi, 1981). On day 44, GI values reached 112.65, 145.26, and 155.52% in T3, T4, and T5, respectively (Supplementary Figure S2e). Factorial analysis showed that both SSP and SynCom significantly increased GI, with a significant SSP × SynCom interaction (*P* < 0.01). CEC increased progressively in all treatments during vermicomposting. On day 44, CEC was significantly higher in T2 than in T1, indicating enhanced humification following cattle manure addition, while T4 showed the highest final CEC among T2-T5 (Supplementary Figure S2d). Because CEC reflects both humification and the water- and nutrient-retention capacity of vermicompost (Gong et al., 2019), these results suggest that combined substrate use and SynCom inoculation promoted humus formation. SynCom inoculation may have promoted humification through enhanced lignocellulose degradation and interactions with indigenous microorganisms. As reported in previous studies, the increase in CEC may be associated with the greater formation of oxygen-containing functional groups (Luo et al., 2026). Overall, SSP and SynCom, alone or in combination, promoted vermicompost maturation, reduced phytotoxicity, and improved the water- and nutrient-retention capacity of the final products. However, these maturity indicators do not establish hygienic safety; future studies should therefore quantify relevant pathogen indicators to determine compliance with commercial fertilizer hygiene requirements, such as those specified in Regulation (EU) 2019/1009 (European Parliament and Council of the European Union, 2019).

### 3.3 Effect of SSP and lignocellulose-degrading SynComs on total organic carbon, total nitrogen, and their mineralized forms

**FIGURE 2:**
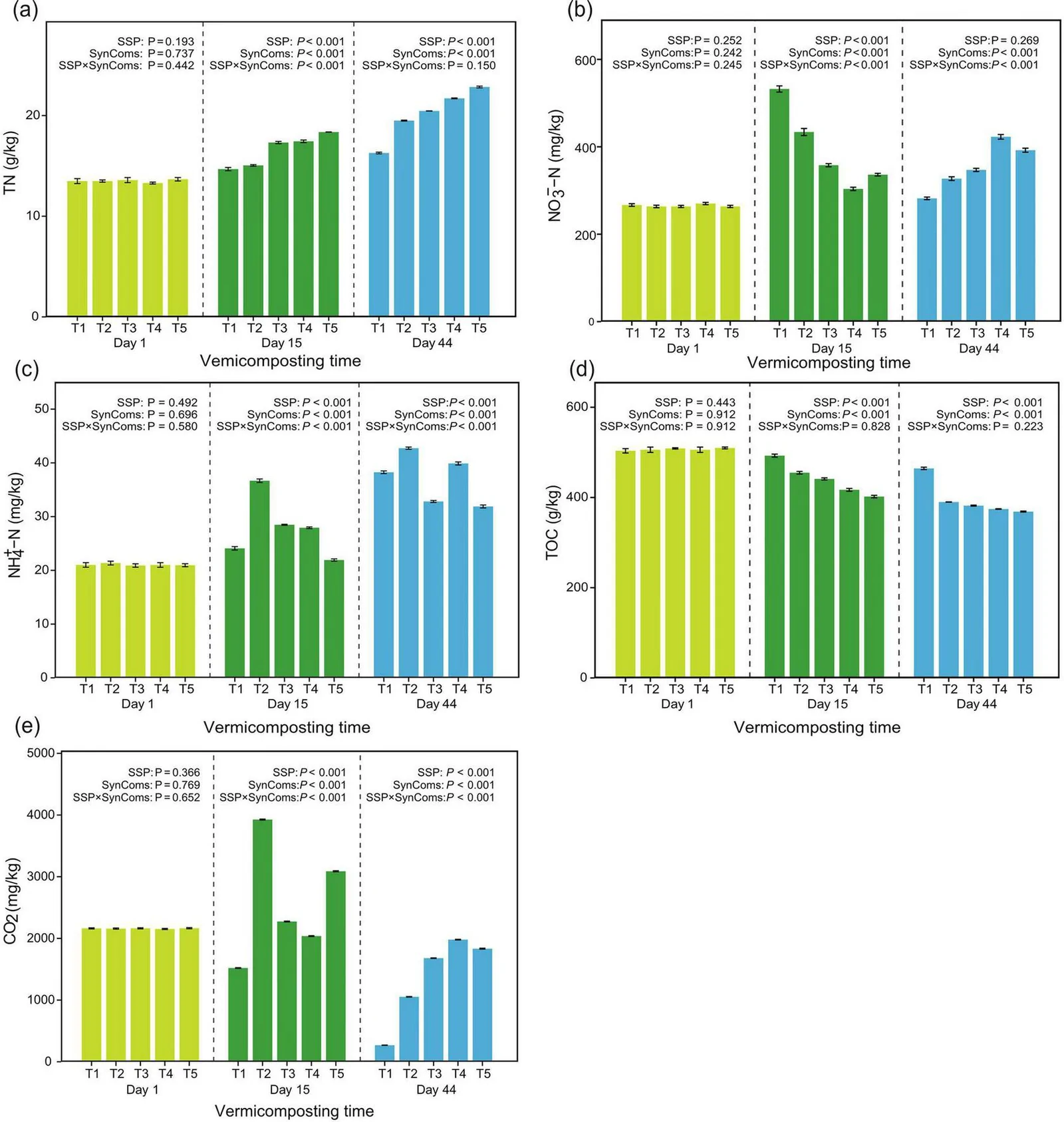
Effects of SSP and/or SynComs inoculation on the total organic carbon, total nitrogen, and their different mineralized forms during vermicomposting. **(a)** The total organic carbon content; **(b)** the CO_2_ concentration; **(c)** the total nitrogen content; **(d)** the ammonium nitrogen content; **(e)** the nitrate nitrogen content. Repeated-measures ANOVA was performed to evaluate the individual and interactive effects of SSP and SynComs on each parameter in treatments T2–T5 at days 1, 15, and 44 of vermicomposting.

In the vermicomposting reactors, TN was primarily composed of inorganic nitrogen, specifically NH_4_^+^-N and NO_3_^–^-N. Relative to the initial substrates, TN increased 1.21-fold in T1 and 1.45–1.47-fold in T2-T5. On day 44, TN contents in T3-T5 were significantly higher than those in T1 and T2. Both SSP and SynComs significantly increased TN (*P* < 0.001), with no significant interaction between them (Figure 2a). Throughout the process, NO_3_^–^-N remained approximately one order of magnitude higher than NH_4_^+^-N. On day 15, T1 showed the highest NO_3_^–^-N content (532.31 mg/kg), 18.50–42.95% higher than those in T2–T5, whereas its NH_4_^+^-N content was 16.18% lower than the mean of T2-T5 (Figures 2b,c). This may be attributed to enhanced ammonification following C/N adjustment by cattle manure, while elevated ammonia levels can suppress nitrifying bacteria (Yang et al., 2024). The relatively high initial NO_3_^–^-N content was likely associated with raw-material properties and storage conditions (Gong et al., 2019).

During vermicomposting, NO_3_^–^-N decreased by 47.02, 24.61, and 3.03% in T1, T2, and T3, respectively, but increased by 28.14 and 14.26% in T4 and T5. In contrast, NH_4_^+^-N increased by 13.21–36.99% in all treatments (Figures 2b,c). Cattle manure increased NH_4_^+^-N by 52.20 and 11.71% on days 15 and 44, respectively, and increased NO_3_^–^-N by 15.99% on day 44. Compared with T2, T3–T5 had 22.36–40.24% and 6.65–25.37% lower NH_4_^+^-N on days 15 and 44, respectively, but 6.11–29.28% higher NO_3_^–^-N on day 44. SynComs significantly increased NO_3_^–^-N and decreased NH_4_^+^-N, whereas SSP significantly decreased NH_4_^+^-N. Their interaction further increased NO_3_^–^-N and decreased NH_4_^+^-N (*P* < 0.01). SSP may enhance NH_4_^+^-N retention through ammonium phosphate formation and pH reduction, thereby limiting NH_3_ volatilization (Wang et al., 2026). Overall, SSP and SynComs enhanced N retention, with T4 showing the highest NO_3_^–^-N content, suggesting a stronger effect of SynComs on nitrification and N conservation (Wang Y. et al., 2025).

TOC loss mainly resulted from CO_2_ release and the consumption of soluble organic C by earthworms and microorganisms (Ding et al., 2026). TOC decreased by 2.13–21.14% during pre-composting and by a further 5.74–14.27% during vermicomposting (Figure 2d). At both stages, T2 had significantly lower TOC than T1. Compared with T2, SSP or SynComs alone significantly reduced TOC, whereas their combined addition had no significant effect. No significant SSP × SynCom interaction was detected.

CO_2_ release, an indicator of aerobic biological activity (Wang et al., 2026), decreased by 42.28% in T1 during pre-composting but increased by an average of 23.71% in T2–T5 relative to their initial levels (Figure 2e). During vermicomposting, CO_2_ release decreased by 2.83–82.32%. T2 showed significantly higher CO_2_ release than T1, while SSP and SynComs, individually or in combination, further increased CO_2_ release, with the highest value observed in T4. However, SSP weakened the stimulatory effect of SynComs in the combined treatment, possibly because SSP-induced decreases in pH and C/N selectively inhibited certain bacterial groups and reduced microbial respiration during the later stage (Ajibade et al., 2020).

### 3.4 Effect of SSP and lignocellulose-degrading SynComs on changes in available nutrients and humic composition

**FIGURE 3:**
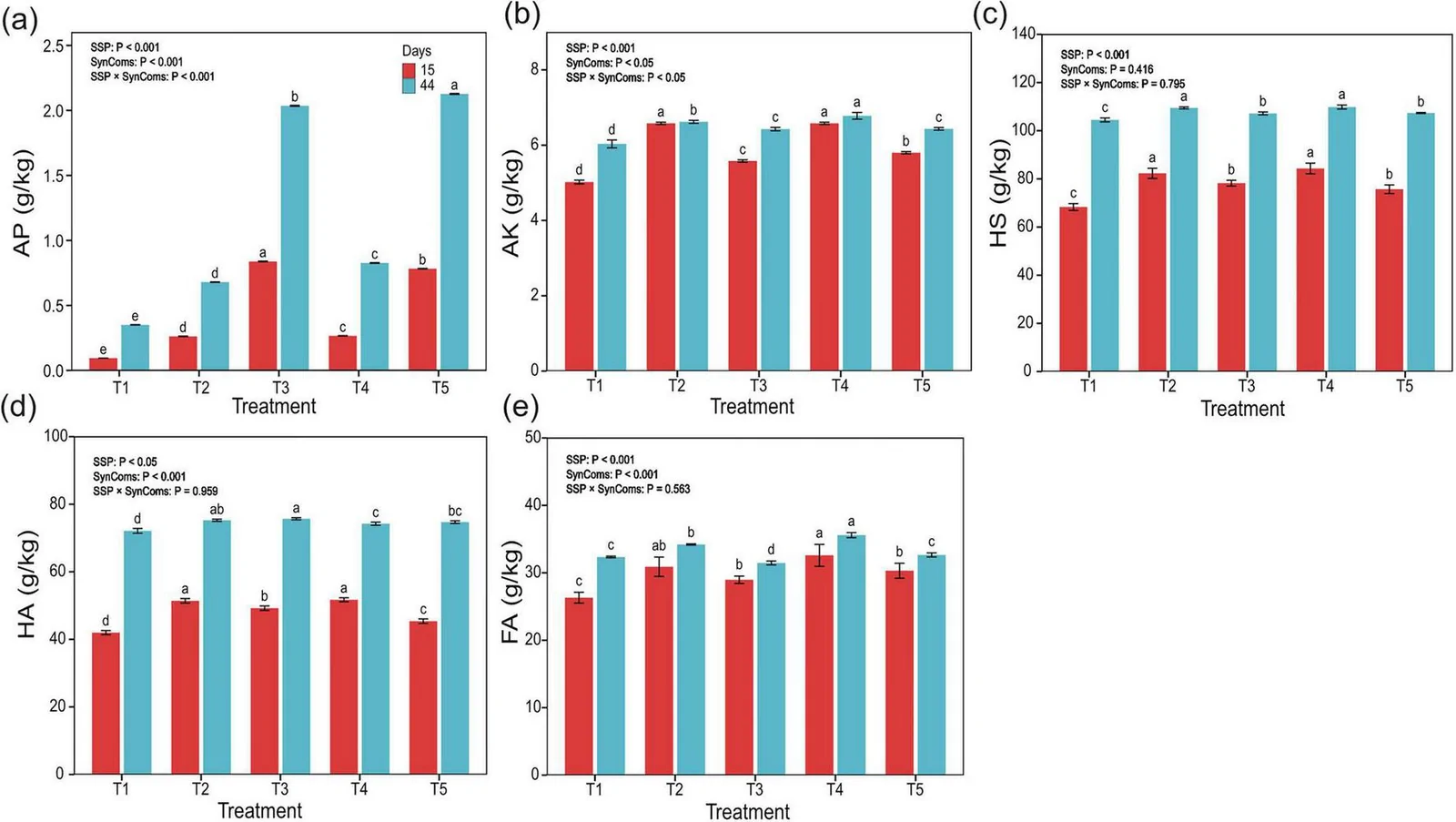
Effects of SSP and/or SynCom inoculation on available nutrients and humic substance fractions during vermicomposting. **(a)** Available phosphorus content; **(b)** available potassium content; **(c)** total humic substance content; **(d)** humic acid content; and **(e)** fulvic acid content. Different lowercase letters indicate significant differences among treatments at the same sampling time point based on one-way ANOVA followed by LSD test (*P* < 0.05). Repeated-measures ANOVA was performed for treatments T2–T5 to evaluate the main effects of SSP, SynCom, and their interactions, on each parameter.

Available phosphorus (AP) and potassium (AK) increased during pre-composting and vermicomposting, likely due to mass loss, organic matter degradation, and the absence of leachate loss in the closed system (Ding et al., 2026). Relative to the initial substrates, AP and AK in the final products increased by an average of 93.43 and 36.59%, respectively (Figures 3a,b). On day 44, cattle manure increased AP and AK by 93.80 and 9.72%, respectively, compared with green waste alone. Among T2-T5, AP in T3–T5 was 21.65–213.03% higher than that in T2, whereas only T4 showed a slight increase in AK (2.42%). Factorial analysis showed that both SSP and SynComs significantly increased AP, with a significant SSP × SynCom interaction (*P* < 0.01). For AK, only SSP had a significant main effect, while the interaction was also significant (*P* < 0.001). These results suggest that SSP enhanced P availability mainly through chemical processes, whereas SynComs promoted P and K transformation through microbial activities, including phosphatase and organic acid production (Zhao et al., 2023).

Humic acid (HA) and fulvic acid (FA) are the major components of humic substances (HS) (Feng et al., 2024). HS increased in all treatments during vermicomposting, with HA remaining the dominant fraction and showing a similar temporal trend. On day 44, cattle manure increased HS, HA, and FA by 4.97, 4.63, and 5.74%, respectively, compared with green waste alone (Figures 3c–e). Among T2–T5, T4 increased HS and FA by 0.33 and 4.05%, respectively, whereas T3 increased HA by 0.59% relative to T2. FA remained lower than HA throughout vermicomposting, likely reflecting the microbial conversion of labile organic fractions into more stable humic structures during maturation (Feng et al., 2024). Factorial analysis showed that SynComs significantly increased HA and FA, whereas SSP significantly decreased HS and HA. Significant SSP × SynCom interactions were observed for HA and FA (*P* < 0.01). T4 showed the highest final HS content (109.83 ± 0.84 g/kg), suggesting that SynCom-enhanced lignocellulose degradation promoted the release of humic precursors and subsequent humification (Chen et al., 2025b).

### 3.5 Effect of SSP and lignocellulose-degrading SynComs on the quality of the vermicompost

Principal component analysis (PCA) was performed using the 18 key quality indicators of the final vermicompost products described above. The effects of SSP and/or SynComs on vermicompost quality were evaluated by ranking the principal component scores and the comprehensive evaluation index. A 5 × 18 matrix was constructed based on the five treatments and 18 quality indicators. PCA was then used for dimensionality reduction, and a limited number of principal components (PCs) explaining more than 85% of the total variance were extracted. The expression of each principal component was constructed by multiplying the eigenvector coefficients by the corresponding standardized variables. PC scores were then calculated, and a comprehensive evaluation index (F) was derived to rank the quality of the different treatments. The PCA results showed that the three extracted principal components cumulatively explained 98.74% of the total variance (Supplementary Table S5). PC1 explained 69.27% of the variance and was mainly driven by TN. PC2 explained 20.29% and was primarily associated with pH, whereas PC3 explained 9.18% and was mainly influenced by GI. The scores of each PC, calculated based on the standardized variables and scoring coefficient matrix, were weighted and integrated to obtain the comprehensive evaluation coefficients (Supplementary Tables S6–S8). The comprehensive quality ranking of the treatments was T4 > T5 > T3 > T2 > T1, indicating that T4 produced the highest-quality vermicompost product. Interestingly, the highest comprehensive quality score was observed in the SynCom treatment rather than the combined SSP + SynCom treatment, suggesting that additional phosphate amendment did not further improve the overall benefits of SynCom inoculation. This finding indicates that the expected synergistic effect between SSP and SynComs was not fully realized under the present experimental conditions.

### 3.6 Effect of SSP and lignocellulose-degrading SynComs on microbial community during vermicomposting

To elucidate the microbiological mechanisms by which additives enhanced vermicomposting performance, microbial community dynamics were monitored and analyzed at the initial mixing stage (day 0), during pre-composting (days 11 and 15), and at the end of vermicomposting (day 44). NMDS analysis showed that the bacterial community underwent significant succession throughout the composting process, whereas the fungal community showed significant separation only on days 0, 15, and 44 (Supplementary Figure S3). A distinct bacterial community was observed on day 44, indicating continuous microbial succession during the formation and aging of vermicompost aggregates and the depletion of substrate nutrients. This finding is consistent with previous reports that bacterial community succession during vermicomposting is primarily driven by changes in nutrient pools (Medina-Sauza et al., 2019).

**FIGURE 4:**
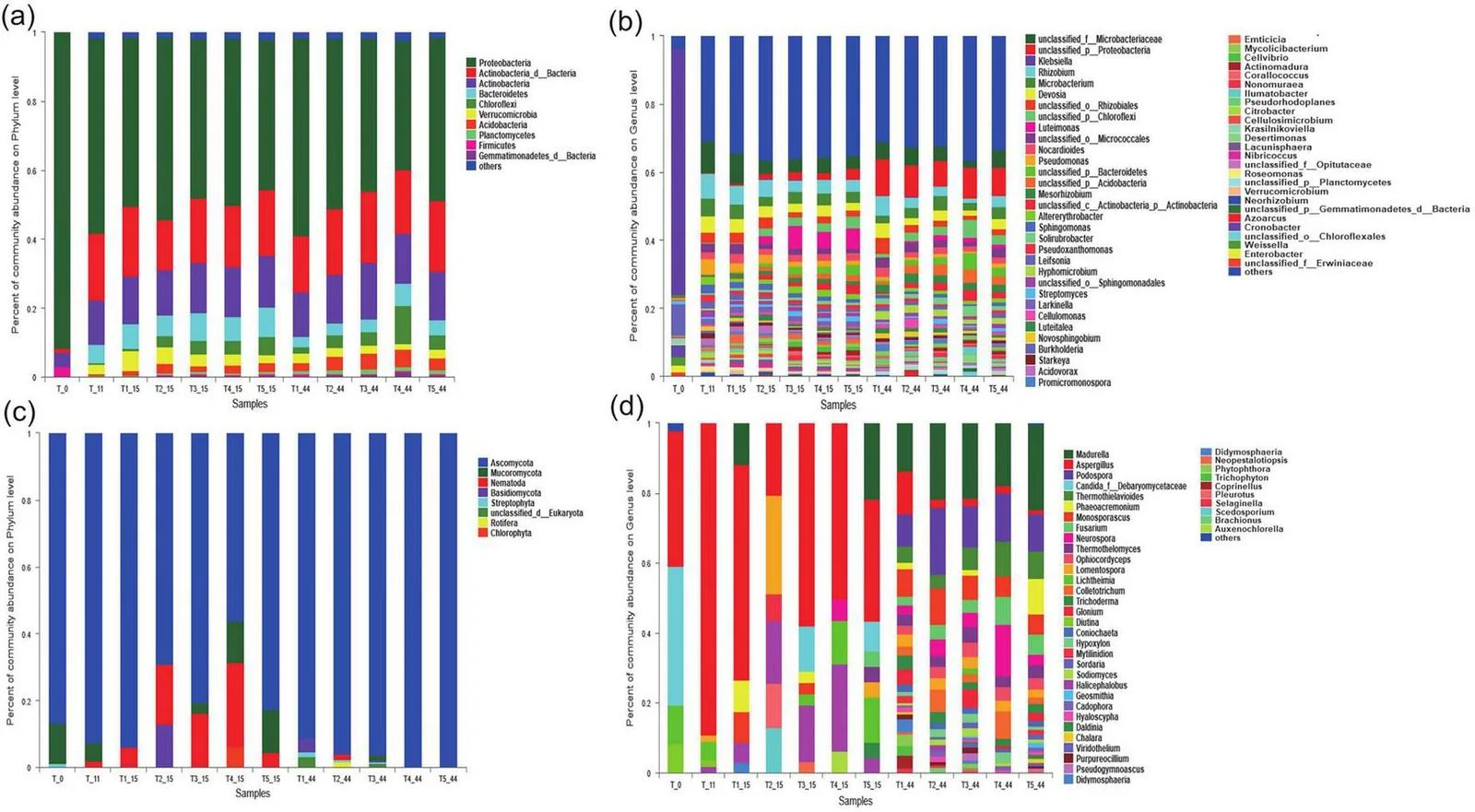
Effects of SSP and/or SynComs inoculation on bacterial and fungal community composition during vermicomposting; **(a)** relative abundance of bacterial communities at the phylum level; **(b)** relative abundance of fungal communities at the phylum level; **(c)** relative abundance of bacterial communities at the genus level; **(d)** relative abundance of fungal communities at the genus level.

Phylum-level analysis showed that the dominant bacterial taxa mainly included *Proteobacteria*, *Actinobacteria*_*d*__*Bacteria*, *Actinobacteria*, *Bacteroidetes*, *Verrucomicrobia*, *Chloroflexi*, *Planctomycetes*, and *Acidobacteria* (Figure 4a). The bacterial community composition was generally consistent with that previously observed in the gut of *Eisenia fetida* and earthworm casts, although the relative abundances of dominant phyla were affected by substrate type (Aira et al., 2016; Nechitaylo et al., 2010; Rattray et al., 2010; Jin et al., 2025). As composting proceeded, the relative abundance of *Proteobacteria* continuously decreased, whereas those of *Actinobacteria*_*d*__*Bacteria* and *Actinobacteria* gradually increased. On day 44, the relative abundance of *Actinobacteria* in the combined-substrate treatments (T2–T5) was higher than that in T1, indicating that the community gradually succeeded from copiotrophic to oligotrophic bacteria. This pattern is consistent with microbial succession during earthworm cast maturation (Aira et al., 2019).

Genus-level analysis further showed that the relative abundances of *Microbacterium*, *Rhizobium*, *Pseudomonas*, *Devosia*, *Luteimonas*, and *Pseudoxanthomonas* generally increased during composting (Figure 4c). Specifically, the relative abundance of *Luteimonas* in the additive treatments (T3–T5) on days 15 and 44 was 4.9- and 2.7-fold higher than that in T1 and T2, respectively. This genus is typically enriched in NO_3_^–^-rich and aerobic environments and is associated with functions such as promoting NO_2_^–^ oxidation, secreting chitinase, facilitating carbon degradation, and enhancing nitrogen retention (Medina-Sauza et al., 2019). This is consistent with the finding of the present study that additive treatments significantly increased NO_3_^–^_–_N content.

The fungal community was dominated by *Ascomycota* (60.77–70.72%), *Mucoromycota* (24.18–36.46%), *Basidiomycota* (0.32–12.00%), and *Chytridiomycota* (0.03–1.25%) (Figure 4b). On day 44, the relative abundance of *Ascomycota* in the combined-substrate treatments (T2–T5) was 3–13% higher than that in T1, suggesting a potentially greater capacity for lignocellulose degradation. SSP addition reduced the relative abundance of *Mucoromycota*; on day 44, this phylum decreased by 2.30 and 1.23% in T3 and T5, respectively, whereas it increased by 1.00 and 1.40% in T2 and T4, respectively.

Genus-level analysis showed that the initial materials were dominated by *Candida_f_Debaryomycetaceae*, *Lichtheimia*, *Aspergillus*, and *Rhizopus* (Figure 4d). After pre-composting, the relative abundances of *Aspergillus* and *Lichtheimia* decreased by 9–23% and 10–15%, respectively, likely because of the depletion of readily available carbon required for their initial growth (Finore et al., 2023). On day 44, *Aspergillus* remained abundant in T1 (18.0%) but decreased to approximately 4.2% in T2-T5. Its abundance continued to decline despite SynCom supplementation after earthworm addition, which may be associated with altered microbial competition and resource utilization during vermicomposting. In particular, enrichment of actinomycetes in earthworm casts may suppress *Aspergillus* through the production of antifungal metabolites (Jayasinghe and Parkinson, 2009). Gut-mediated homogenization may further stabilize indigenous microbial communities and limit the colonization of exogenous SynComs, consistent with previous observations that inoculated microorganisms can fail to establish because of competition from stimulated indigenous populations (Čaušević et al., 2024). Although *Aspergillus* abundance was lower in the combined-substrate treatments than in the single-substrate treatment, its species- and strain-dependent biosafety remains relevant (Keller et al., 1994). Therefore, strain-level biosafety assessment is recommended before large-scale application of the SynCom to minimize potential toxigenic or pathogenic risks (Cary et al., 2009).

### 3.7 Effect of SSP and lignocellulose-degrading SynComs on the genetic potential of vermicomposting microbial communities in carbohydrate degradation function

**FIGURE 5:**
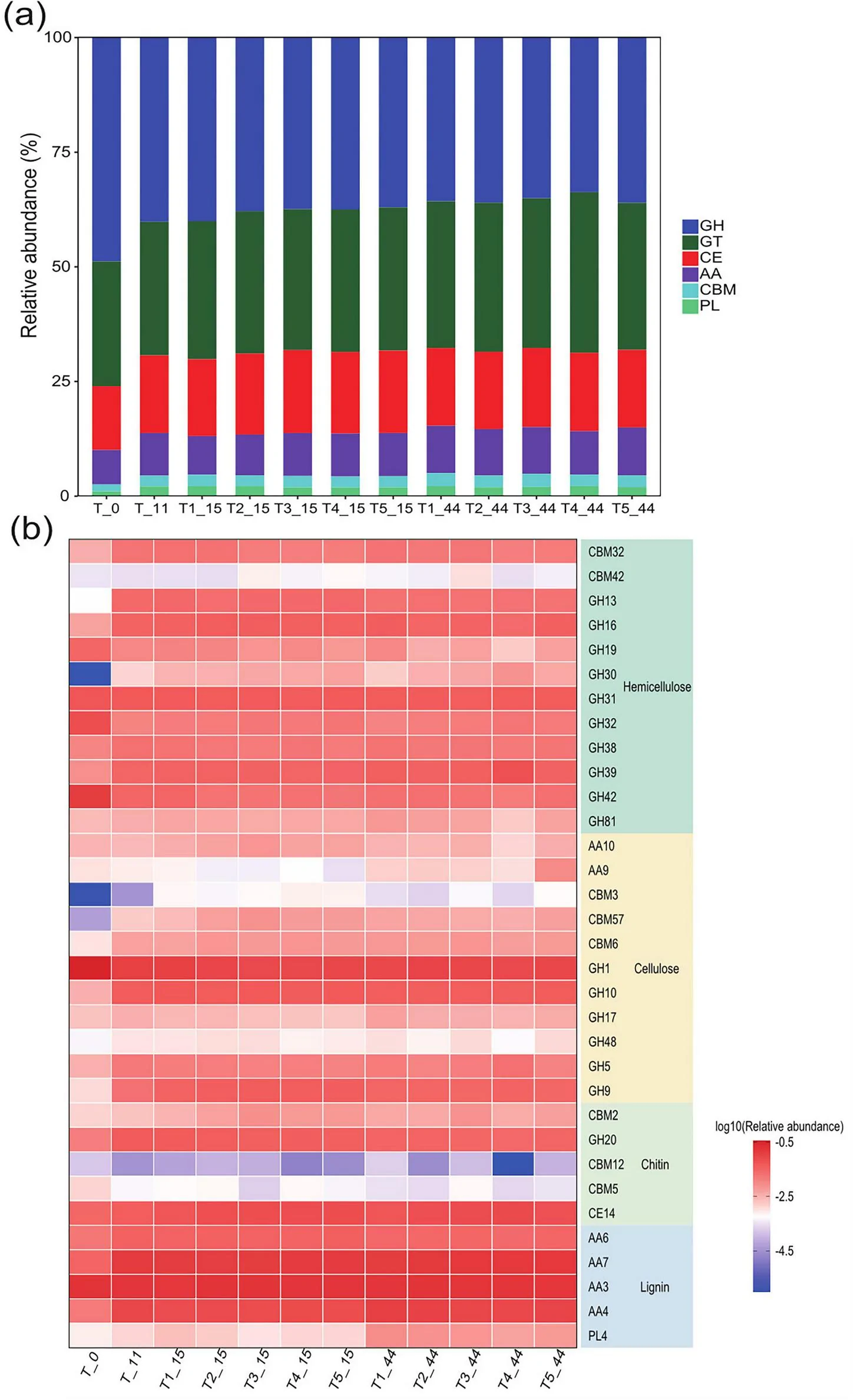
Effects of SSP and/or SynComs inoculation on the abundances of lignocellulose degradation-related functional genes and nitrogen-cycling functional genes; **(a)** relative abundances of the six major CAZyme classes in the CAZy database; **(b)** relative abundances of the top 33 most abundance CAZyme families.

At the CAZyme class level, glycoside hydrolases (GHs, 33.71–48.80%), glycosyltransferases (GTs, 27.21–35.00%), carbohydrate esterases (CEs, 13.91–18.09%), and auxiliary activities (AAs, 7.51–10.43%) were more abundant than polysaccharide lyases (PLs, 1.07–2.19%) and carbohydrate binding modules (CBMs, 1.50–2.86%) (Figure 5a), indicating strong microbial potential for lignocellulose degradation and subsequent transformation of degradation products (Zhang et al., 2025). During vermicomposting, GHs abundance consistently decreased, whereas GTs and CBMs increased across all treatments. On day 15, cattle manure addition increased AAs and CEs by 5.59 and 5.37%, respectively, but decreased GHs and PLs by 5.49% and 1.37% compared with green waste alone. Among T2–T5, T3–T5 similarity showed higher AAs (5.18–5.74%) and CEs (0.85–2.35%) but lower GHs (0.95–2.17%) and PLs (11.11–11.57%) than T2. On day 44, cattle manure addition increased only under the SSP-alone and SynCom-alone treatments, by 0.31 and 7.63%, respectively, while CEs and PLs in T3–T5 were 0.59–2.55% and 1.525–11.68% higher than those in T2. The increase in CEs may enhance hemicellulose deacetylation and accessibility to hydrolytic enzymes, thereby facilitating lignocellulose degradation (Zhang et al., 2025; Chen et al., 2025b). Notably, the pronounced GTs enrichment in T4 suggests that SynCom inoculation may enhance the glycosylation and stabilization of lignin-derived phenolics, potentially promoting microbial residue accumulation and humus formation (Chen et al., 2025a).

Specific CAZyme family analysis further characterized changes in the genetic potential associated with lignocellulose degradation. The additive treatments (T3-T5) significantly enriched the AA3 and AA7 families, which encode auxiliary activity enzymes (Figure 5b). These oxidative enzymes can act synergistically with GHs to promote lignocellulose decomposition. The relative abundances of key cellulase-related families (GH5 and GH10) and hemicellulase-related families (GH32 and GH39) were higher in the amendment treatments, suggesting an increased genetic potential for oxidative and hydrolytic processes involved in lignocellulose degradation. These results explain the enhanced lignocellulose degradation observed in T3-T5 at the functional gene level and suggest that SSP and/or SynComs can improve substrate-directed degradation efficiency by reshaping the microbial community and its CAZyme functional potential (Chen et al., 2025b).

### 3.8 Effect of SSP and lignocellulose-degrading SynComs on the genetic potential of microbial communities in nitrogen cycling function during vermicomposting

**FIGURE 6:**
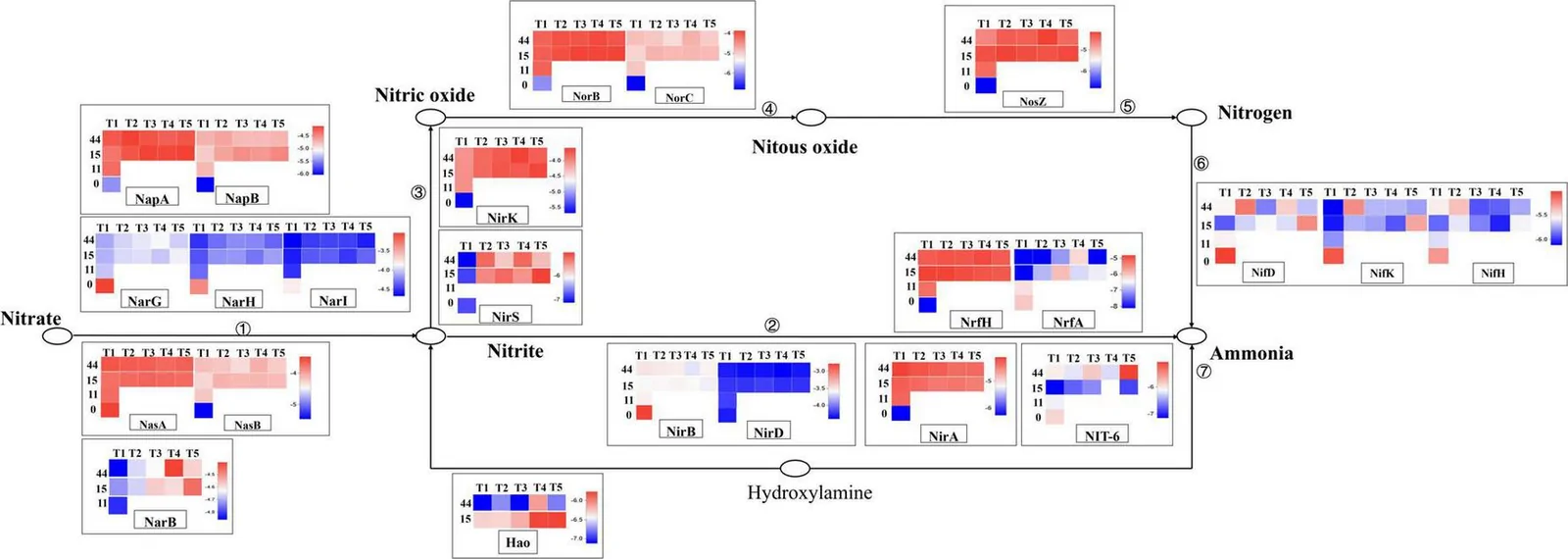
Effects of SSP and/or SynComs inoculation on the abundances of nitrogen-cycling functional genes.

Five major nitrogen-cycling functions were identified in the vermicomposting system, including nitrogen fixation (*nifH, nifD*, and *nifK*), nitrification-related hydroxylamine oxidation (*hao*), denitrification (*nirK, nirS, norB, norC*, and *nosZ*), dissimilatory nitrate reduction to ammonium (*narG, narH, narI, napA, napB, nrfA*, and *nrfH*), and assimilatory nitrate reduction to ammonium (*nasA, nasB, narB, nirB, nirD*, and *NIT-6*) (Figure 6). The abundance profiles of nitrogen cycling-related functional genes in the day 0 samples differed markedly from those at subsequent stages, with the dissimilatory membrane bound nitrate reductase genes *narGHI* showing the highest relative abundances. As vermicomposting progressed, the abundances of *narGHI* in T1 continuously decreased and consistently followed the order *narG* > *narH* > *narI*. In contrast, the average abundances of *narGHI* and denitrification related genes in T2-T5 were generally higher than those in T1, suggesting that co-vermicomposting green waste with cattle manure may enhance the genetic potential for microbial nitrate reduction and denitrification. Taxonomic annotation against the NR database further showed that the potential functional microbial communities associated with denitrification genes exhibited higher α diversity, with generally higher Shannon diversity and Chao richness indices than the microbial communities associated with the nitrification related *hao* gene. Meanwhile, the overall relative abundance of denitrification related functional genes was also higher than that of *hao* (Figure 6; Supplementary Table S9), with *nirK* being more abundant than *nirS*. Within the assimilatory nitrate reduction pathway, the abundances of genes involved in nitrate reduction to nitrite followed the order *nasA* > *nasB* > *narB*, whereas those involved in nitrite reduction to ammonium followed the order *nirB* > *nirD* > *nirA* > *NIT-6*. In addition, compared with T1, the average abundance of the nitrogen fixation genes *nifHDK* in T2-T5 increased by 51.75% on day 15 but decreased by 10.00% by day 44, indicating a stage dependent effect of the combined substrate on the genetic potential for nitrogen fixation.

Changes in nitrogen cycling related functional genes were broadly consistent with the transformation patterns of inorganic N among treatments. During pre-composting, mixing cattle manure with green waste promoted organic N mineralization and NH_4_^+^-N formation, whereas NO_3_^–^-N remained the predominant inorganic N form across treatments during the subsequent vermicomposting stage. Factorial analysis further showed that SynComs significantly increased NO_3_^–^-N content while decreasing NH_4_^+^-N content, whereas SSP mainly decreased NH_4_^+^-N content. Their interaction simultaneously promoted NO_3_^–^-N accumulation and decreased NH_4_^+^-N content (*P* < 0.01; section 3.3). These changes were consistent with the enhanced functional potential of the nitrification related *hao* gene under SynCom inoculation, suggesting that lignocellulose degrading SynComs may alter carbon substrate availability and microbial metabolic activity, thereby favoring N transformations associated with hydroxylamine oxidation. However, the *amoA* gene of ammonia-oxidizing bacteria (AOB) was not detected in this study; therefore, the abundance of *hao* together with changes in inorganic N forms is insufficient to demonstrate enhancement of the complete ammonia oxidation process. Overall, our results support an enhanced genetic potential for nitrification related hydroxylamine oxidation following SynCom inoculation, whereas its effect on the complete ammonia oxidation process requires further verification through quantitative analyses of *amoA* genes from ammonia-oxidizing archaea (AOA), AOB, and complete ammonia oxidizers (comammox), together with analyses of their transcriptional activity.

Compared with the relatively limited genetic signal associated with nitrification, a more complete suite of denitrification related functional genes, including *nirK/nirS, norB/norC*, and *nosZ*, was detected, indicating a substantial genetic potential for denitrification in the vermicomposting system. The average abundances of denitrification related genes and *narGHI* were higher in T2-T5 than in T1, suggesting that co-vermicomposting green waste with cattle manure may provide more favorable ecological conditions for nitrate-reducing and denitrifying microorganisms. This response may be associated with increased availability of utilizable organic carbon and greater spatial heterogeneity in redox conditions within the combined substrate (Cao et al., 2021; Yang et al., 2024). Furthermore, by promoting lignocellulose degradation and microbial respiration, SynComs may increase the supply of readily available carbon substrates and oxygen consumption, thereby creating localized low oxygen microsites favorable for heterotrophic nitrate-reducing and denitrifying microorganisms (Luo et al., 2026). The higher abundance of *nirK* than *nirS* further suggests that *nirK*-type nitrite-reducing microorganisms may contribute more prominently to the denitrification potential of this system. Nevertheless, functional gene abundance represents metabolic potential rather than actual process rates; therefore, these results cannot be used to directly infer increases in denitrification rates or N_2_O emissions.

The concurrent occurrence of *nirK/nirS, norB/norC*, and *nosZ* along the denitrification pathway further indicates that this system possesses genetic potential for both N_2_O production and reduction. The higher abundance of *nosZ* in T2–T5 than in T1 suggests that co-vermicomposting green waste with cattle manure may enhance the microbial functional potential for N_2_O reduction to N_2_. Meanwhile, the relatively high abundances of *nirK/nirS* and *norB/norC* indicate substantial genetic potential for the sequential reduction of NO_2_^–^ to NO and subsequently to N_2_O. Therefore, the effects of SSP and SynComs on net N_2_O emissions may depend on the balance between N_2_O production and *nosZ* mediated N_2_O reduction, which is further regulated by utilizable carbon availability, NO_3_^–^-N supply, pH, and local oxygen conditions (Aldossari and Ishii, 2021; Yang et al., 2024). Considering the effects of SynComs on organic matter decomposition, microbial respiration, NO_3_^–^-N accumulation, and nitrogen cycling related functional genes observed in this study, SynCom inoculation may promote N transformation while strengthening the spatial coupling between nitrification and denitrification within the vermicomposting substrate. Future studies integrating N_2_O flux measurements, expression analyses of key functional genes, and microscale oxygen measurements are needed to verify these mechanisms (Zhao et al., 2023). From a practical perspective, maintaining adequate aeration and appropriate substrate structure may help minimize the formation of excessively oxygen limited microsites, thereby retaining the benefits of SynComs for lignocellulose degradation and N transformation while reducing the potential risk of N loss through denitrification. A conceptual overview of the experimental design, amendment strategies, and the potential effects of SSP and lignocellulose-degrading SynComs on lignocellulose degradation, microbial succession, and carbon- and nitrogen-cycling functional potential during co-vermicomposting is presented in Supplementary Figure S4.

## 4 Conclusion

This study demonstrates that co-vermicomposting of cattle manure and highly lignified green waste is a feasible strategy for urban organic waste valorization. Compared with single-substrate vermicomposting, co-vermicomposting promoted lignocellulose degradation and improved overall vermicomposting performance. Among the co-vermicomposting treatments, SynCom inoculation alone produced the greatest overall improvement, characterized by enhanced lignocellulose degradation and humification, changes in nitrogen transformation indicators, and increased microbial carbon- and nitrogen-cycling potential. Although SSP alone affected several physicochemical and nitrogen-related properties, its interactions with SynComs were process dependent, and SSP weakened several stimulatory effects of SynCom inoculation, particularly those related to microbial enzyme activities and overall vermicompost quality. Thus, the combined application of SSP and SynComs did not show a consistent advantage over SynCom inoculation alone. Under the experimental conditions of this study, SynCom inoculation alone showed the greatest potential as a bioaugmentation strategy, although pathogen related indicators, fungal metabolites, and strain level biosafety should be further evaluated before large scale application.

## Data Availability

The datasets presented in this study can be found in online repositories. The names of the repository/repositories and accession number(s) can be found in the article/Supplementary material.
