# Supplementary material for: A lignocellulose-degrading synthetic microbial community alters physicochemical properties, microbial community structure, and functional gene potential during green waste-cattle manure co-vermicomposting
Source: Front Microbiol. 2026 Sep 9;17:1927926. doi: 10.3389/fmicb.2026.1927926 (PMC13597777; doi:10.3389/fmicb.2026.1927926)
Supplement: Supplementary file 1 [file Table_1.docx]

Supplementary Material

# 1 Supplementary Figures and Tables

## 1.1 Supplementary Figures

**Supplementary Figure 1.** Taxonomic dentification and lignocellulose-degrading characteristics of the selected microbial stains. (a) Colony morphology of strain 2 (Bacillus sp.) and its phylogenetic tree constructed based on 16S rRNA gene sequences (original isolated code: CL-2). (b) Colony morphology of strain 5 (Bscillus sp.) and its phylogenetic tree constructed based on 16S rRNA gene sequences (original isolated code: lou2). (c) Colony morphology of strain 11 (Aspergillus sp.) and its phylogenetic tree constructed based on ITS sequences (original isolated code: ITS1). (d-f) MnP and LiP production capacities of strains 2, 5, 11, and strain A. (g) Lac production capacity of strain 11. (h) Cellulase production capacities of strains 2, 5, 11 and strain A.

**Supplementary Figure 2.** Effects of SSP and SynComs inoculation on maturity-related indicators during vermicomposting. (a) the pH value; (b) the electrical conductivity value; (c) the carbon-to-nitrogen ratio value; (d) the cation exchange capacity value; (e) the germination index value. Different lowercase letters indicate significant differences among treatments at each sampling time point according to the least significant difference (LSD) test (P < 0.05). The repeated-measures ANOVA was performed using treatment T2-T5 to evaluate the main effects of SSP, SynComs, and composting tome, as well as their interaction effect on maturity-related indicators during vermicomposting. EC: electrical conductivity; C/N: carbon-to-nitrogen ratio; CEC: cation exchange capacity. GI: germination index. T1-T5 are described in Section 2.3.

**Supplementary Figure 3.** Effects of SSP and/or SynCom inoculation on microbial diversity during vermicomposting. (a) Non-metric multidimensional scaling (NMDS) based on Bray-Curtis dissimilarities showing microbial β-diversity and differences in bacterial community successions trajectories among treatments. (b) Differents in bacterial community succession trajectories among treatments on days 11, 15, and 44 of vermicomposting. (c) Differences in bacterial community succession trajectories among treatments on days 0, 11, 15, and of vermicomposting.

**Supplementary Figure 4.** Graphical abstract illustrating the experimental design and proposed mechanisms underlying the effects of SSP and lignocellulose-degrading SynComs during green waste–cattle manure co-vermicomposting.

**Supplementary Table 1.** Basic physicochemical properties of compost raw materials.

|  | Green waste | Cattle manure |
| --- | --- | --- |
| pH | 7.0 ± 0.03 | 7.65 ± 0.1 |
| EC(mS·cm^-1^) | 1.03 ± 0.04 | 3.17 ± 0.05 |
| Total organic carbon (g·kg^-1^) | 506.73 ± 2.36 | 238.23 ± 0.48 |
| Total nitrogen (g·kg^-1^) | 12.39 ± 0.20 | 31.48 ± 0.15 |
| Lignin content (%) | 26.14 ± 0.22 | 8.70 ± 0.07 |
| Cellulose content (%) | 38.87 ± 0.18 | 17.70 ± 0.67 |
| Available phosphorus (mg·kg^-1^) | 146.36 ± 0.98 | 478.55 ± 6.9 |
| Available potassium (g·kg^-1^) | 3.66 ± 0.06 | 12.11 ± 0.05 |
| Ammonium nitrogen (mg·kg^-1^) | 26.84 ± 0.1 | 90.24 ± 0.93 |
| Cation exchange capacity (cmol·kg^-1^) | 23.80 ± 1.4 | 39.82 ± 1.7 |
| Total manganese content (mg·kg^-1^) | 67.24 ± 2.6 | 166.30 ± 0.37 |
| Total iron content (mg·kg^-1^) | 2.40 ± 0.03 | 3116 ± 42 |
| Total copper content (mg·kg^-1^) | 174.83 ± 3.08 | 18.55 ± 0.18 |
| Total zinc content (mg·kg^-1^) | 284.52 ± 3.40 | 105.49 ± 0.51 |
| Total chromium content (mg·kg^-1^) | 0.23 ± 0.04 | 3.32 ± 0.1 |
| Total lead content (mg·kg^-1^) | 10.70 ± 1.5 | 28.90 ± 1.31 |
| Total nickel content (mg·kg^-1^) | 1.34 ± 0.03 | 36.47 ± 1.45 |

Values are presented as mean ± SD (n = 3).

**Supplementary Table 2.** Mixture design matrix showing the proportions of four microbial inocula used for optimization of a soild-state fermentation medium.

| Treatment | *No*.A (ml) | *No*.2 (ml) | *No*.11 (ml) | *No*.5 (ml) |
| --- | --- | --- | --- | --- |
| T1 | 1.25 | 1.25 | 1.25 | 1.25 |
| T2 | 0.83 | 0.83 | 1.67 | 1.67 |
| T3 | 0.83 | 1.67 | 0.83 | 1.67 |
| T4 | 0.83 | 1.67 | 1.67 | 0.83 |
| T5 | 1.67 | 0.83 | 0.83 | 1.67 |
| T6 | 1.67 | 0.83 | 1.67 | 0.83 |
| T7 | 1.67 | 1.67 | 0.83 | 0.83 |
| T8 | 5 | 0 | 0 | 0 |
| T9 | 0 | 5 | 0 | 0 |
| T10 | 0 | 0 | 5 | 0 |
| T11 | 0 | 0 | 0 | 5 |
| CK | 0 | 0 | 0 | 0 |

**Supplementary Table 3.** Effects of lignocellulose-degrading microbial inoculation of the activities of major ligninolytic enzymes (LiP, MnP, and Lac) during solid-state fermentation.

| Treatment | 1d | | | 18 d | | | 35 d | | |
| --- | --- | --- | --- | --- | --- | --- | --- | --- | --- |
|  | Lip (U·g^-1^) | Mnp (U·g^-1^) | Lac (U·g^-1^) | Lip (U·g^-1^) | Mnp (U·g^-1^) | Lac (U·g^-1^) | Lip (U·g^-1^) | Mnp (U·g^-1^) | Lac (U·g^-1^) |
| T1 | 238 (7.8)d | 3.8 (0.03)g | 4.9 (0.07)b | 143 (3.1)f | 215 (3.5)e | 3.7 (0.17)e | 382 (3.5)b | 57.5 (1.54)e | 1.35 (0.01)e |
| T2 | 191 (4.0)e | 15.3 (0.3)d | 4.3 (0.16)c | 93 (2.6)g | 245 (2.8)bc | 4.9 (0.11)d | 143 (3.3)g | 23 (1.02)i | 1.62 (0.03)d |
| T3 | 238 (7.3)d | 34.9 (0.94)b | 3.1 (0.19)e | 334 (3.8)c | 157 (1.9)g | 6.2 (0.14)c | 334 (2.9)c | 49.8 (1.2)g | 1.59 (0.11)d |
| **T4** | **334 (3.8)b** | **39.6 (1.37)a** | **8.6 (0.07)a** | **5061 (10.0)a** | **299 (4.2)a** | **19.7 (1.27)a** | **573 (4.7)a** | **153.2 (2.76)a** | **4.93 (0.03)a** |
| T5 | 49 (4.0)h | 38.3 (0.74)a | 1.2 (0.24)f | 99 (2.0)g | 161 (3.1)g | 4.9 (0.07)d | 239 (3.6)e | 53.6 (1.37)f | 1.4 (0.02)e |
| T6 | 47 (3.9)h | 11.5 (0.45)e | 3.7 (0.07)d | 143 (2.7)f | 188 (4.3)f | 4.7 (0.14)d | 334 (2.9)c | 99.6 (2.4)b | 1.16 (0.04)g |
| T7 | 93 (3.4)g | 23.0 (1.79)c | 4.3 (0.09)c | 1910 (0.7)b | 230 (3.1)d | 9.9 (0.14)b | 239 (2.1)e | 72.8 (1.21)c | 1.01 (0.02)h |
| T8 | 286 (5.1)c | 11.5 (0.52)e | 4.3 (0.16)c | 143 (2.9)f | 241 (2.7)c | 6.2 (0.07)c | 286 (3.4)d | 49.8 (2.20)g | 2.47 (0.04)c |
| T9 | 95 (3.2)f | 11.5 (0.86)e | 4.9 (0.33)b | 157 (5.1)e | 241 (3.7)c | 2.2 (0.04)f | 94 (2.2)h | 42.1 (0.86)h | 1.35 (0.05)e |
| T10 | 812 (4.4)a | 3.8 (0.51)g | 1.2 (0.03)f | 96 (3.7)g | 249 (2.0)b | 2.4 (0.03)f | 48 (2.0)i | 7.7 (0.34)j | 1.23 (0.03)f |
| T11 | 48 (3.3)h | 7.7 (0.34)f | 1.2 (0.05)f | 191 (3.0)d | 69 (3.0)h | 1.2 (0.04)g | 230 (3.6)f | 61.3 (0.71)d | 2.95 (0.01)b |
| CK | 0 (0)i | 0.0 (0)h | 0.0 (0.0)g | 2 (0.03)h | 1.0 (0.2)i | 1.0 (0.02)g | 5 (0)j | 1.1 (0.09)k | 0.97 (0.01)h |

Values are presented as mean ± SD (n = 3). Sampling times of 1d, 8 d, and 35 d correspond to 1, 8, and 35 days after inoculation, respectively. Different lowercase letter within the same column indicates significant differences among treatments (*P* < 0.05).

**Supplementary Table 4.** Residual lignin, cellulose, and hemicellulose contents in the substrate at the end of vermicomposting under different treatments.

| Treatment | Cellulose (%) | Hemicellulose (%) | Lignin (%) |
| --- | --- | --- | --- |
| T1 | 26.70 ± 0.48 a | 12.09 ± 0.49 a | 18.35 ± 0.32 a |
| T2 | 23.65 ± 0.30 b | 10.7 ± 0.28 b | 16.13 ± 0.31 b |
| T3 | 21.59 ± 0.58 c | 8.25 ± 0.31 c | 14.00 ± 0.32 c |
| T4 | 17.33 ± 0.46 d | 6.18 ± 0.10 d | 9.32 ± 0.09 d |
| T5 | 16.52 ± 0.33 e | 5.56 ± 0.09 e | 8.10 ± 0.08 e |

Values are presented as mean ± SD (n = 3). Different lowercase letter within the same column indicates significant differences among treatments (*P* < 0.05).

**Supplementary Table 5.** Principal component analysis results showing the cumulative variance explained by each principal component.

|  | Initial eigenvalue | | | Extracted sum of squared loadings | | |
| --- | --- | --- | --- | --- | --- | --- |
| Principal component No. | Total | Variance contribution (%) | Cumulative variance  contribution (%) | Total | Variance contribution (%) | Cumulative contribution (%) |
|  |  |  |  |  |  |  |
| 1 | 12.468 | 69.268 | 69.268 | 12.468 | 69.268 | 69.268 |
| 2 | 3.652 | 20.288 | 89.556 | 3.652 | 20.288 | 89.556 |
| 3 | 1.652 | 9.179 | 98.735 | 1.652 | 9.179 | 98.735 |
| 4 | 0.228 | 1.27 | 100 |  |  |  |
| 5 | 1.61×10^-15^ | 8.95×10^-15^ | 100 |  |  |  |
| 6 | 1.41×10^-15^ | 7.83×10^-15^ | 100 |  |  |  |
| 7 | 9.00×10^-16^ | 5.00×10^-15^ | 100 |  |  |  |
| 8 | 4.28×10^-16^ | 2.38×10^-15^ | 100 |  |  |  |
| 9 | 3.71×10^-16^ | 2.06×10^-15^ | 100 |  |  |  |
| 10 | 2.09×10^-16^ | 1.16×10^-15^ | 100 |  |  |  |
| 11 | 1.23×10^-16^ | 6.84×10^-16^ | 100 |  |  |  |
| 12 | 1.16×10^-16^ | 6.43×10^-16^ | 100 |  |  |  |
| 13 | -3.31×10^-17^ | -1.84×10^-16^ | 100 |  |  |  |
| 14 | -9.89×10^-17^ | -5.49×10^-16^ | 100 |  |  |  |
| 15 | -2.25×10^-16^ | -1.25×10^-15^ | 100 |  |  |  |
| 16 | -3.32×10^-16^ | -1.84×10^-15^ | 100 |  |  |  |
| 17 | -4.96×10^-16^ | -2.75×10^-15^ | 100 |  |  |  |
| 18 | -7.55×10^-16^ | -4.20×10^-15^ | 100 |  |  |  |

**Supplementary Table 6.** Principal component loading matrix.

| Parameter | Component 1 | Component 2 | Component 3 |
| --- | --- | --- | --- |
| pH | -0.162 | 0.979 | -0.117 |
| EC | 0.982 | -0.059 | 0.046 |
| TN | 0.994 | -0.049 | -0.015 |
| NH_4_^+^-N | -0.373 | 0.924 | -0.07 |
| CO_2_ | 0.978 | -0.018 | -0.05 |
| C/N | -0.965 | -0.005 | 0.251 |
| HS | 0.664 | 0.686 | -0.297 |
| HA | 0.654 | -0.018 | -0.756 |
| CEC | 0.569 | 0.816 | -0.063 |
| The loss of N | -0.9 | 0.346 | 0.247 |
| Cellulase | 0.966 | 0.164 | -0.192 |
| LiP | 0.902 | 0.25 | 0.297 |
| MnP | 0.913 | 0.198 | 0.296 |
| Lac | 0.992 | -0.008 | 0.11 |
| Urease | -0.607 | 0.664 | 0.436 |
| GI | 0.837 | -0.049 | 0.512 |
| Cellulose | -0.969 | 0.029 | -0.244 |
| Lignin | -0.943 | 0.06 | -0.323 |

**Supplementary Table 7.** Eigenvector matrix of the extracted principal components obtained from principal component analysis (PCA).

| Parameter | **t1** | **t2** | **t3** |
| --- | --- | --- | --- |
| pH | -0.05 | 0.51 | -0.09 |
| EC | 0.28 | -0.03 | 0.04 |
| TN | 0.28 | -0.03 | -0.01 |
| NH_4_^+^-N | -0.11 | 0.48 | -0.05 |
| CO_2_ | 0.28 | -0.01 | -0.04 |
| C/N | -0.27 | 0 | 0.2 |
| HS | 0.19 | 0.36 | -0.23 |
| HA | 0.19 | -0.01 | -0.59 |
| CEC | 0.16 | 0.43 | -0.05 |
| The of loss of N | -0.25 | 0.18 | 0.19 |
| Cellulase | 0.27 | 0.09 | -0.15 |
| LiP | 0.26 | 0.13 | 0.23 |
| MnP | 0.26 | 0.1 | 0.23 |
| Lac | 0.28 | 0 | 0.09 |
| Urease | -0.17 | 0.35 | 0.34 |
| GI | 0.24 | -0.03 | 0.4 |
| Cellulose | -0.27 | 0.02 | -0.19 |
| Lignin | -0.27 | 0.03 | -0.25 |

**Supplementary Table 8.** Principal component scores (F1-F3), comprehensive evaluation scores (F), and overall rankings of vermicompost quality under different treatments.

| Treatment | F1 | F2 | F3 | Comprehensive score (F) | Ranking (F) |
| --- | --- | --- | --- | --- | --- |
| T1 | -19.37 | -1.09 | 1.34 | -13.69 | 5 |
| T2 | -3.92 | 3.83 | -1.58 | -2.11 | 4 |
| T3 | 2.07 | -3.49 | -1.92 | 0.56 | 3 |
| T4 | 9.68 | 3.91 | 1.55 | 7.74 | 1 |
| T5 | 11.54 | -3.17 | 0.6 | 7.5 | 2 |

The principal component equations were derived from the variable loading matrix, and the comprehensive evaluation model was established using variance contribution rates derived from the corresponding eigenvalues (Equations 3-1 to 3-4).

F1 = -0.162×ZX1 + 0.982×ZX2 + 0.994×ZX3 - 0.373×ZX4 + 0.978×ZX5 - 0.965×ZX6 + 0.664×ZX7 + 0.654×ZX8 +0.569×ZX9 - 0.900×ZX10 + 0.966×ZX11 + 0.902×ZX12 + 0.913×ZX13 +0.992×ZX14 - 0.607×ZX15 + 0.837×ZX16 - 0.969×ZX17 - 0.943×ZX18 (3-1)

F2 = 0.979×ZX1 - 0.059×ZX2 - 0.049×ZX3 + 0.924×ZX4 - 0.018×ZX5 - 0.005×ZX6 + 0.686×ZX7 - 0.018×ZX8 + 0.816×ZX9 + 0.346×ZX10 + 0.164×ZX11 + 0.250×ZX12 + 0.198×ZX13 - 0.008×ZX14 + 0.664×ZX15 - 0.049×ZX16 + 0.029×ZX17 + 0.06×ZX18 (3-2)

F3 = 0.117×ZX1 + 0.046×ZX2 - 0.015×ZX3 - 0.07×ZX4 - 0.05×ZX5 + 0.251×ZX6 - 0.297×ZX7 - 0.756×ZX8 - 0.063×ZX9 + 0.247×ZX10 - 0.192×ZX11 + 0.297×ZX12 + 0.296×ZX13 + 0.11×ZX14 + 0.436×ZX15 + 0.512×ZX16 - 0.244×ZX17 - 0.323×ZX18 (3-3)

F = [0.69268/ (0.69268 + 0.20288 + 0.09179)] ×F1 + [0.20288/ (0.69268 + 0.20288 + 0.09179)] ×F2 + [0.09179/ (0.69268 + 0.20288 + 0.09179)] ×F3 = 0.701554667×F1 + 0.205479313×F2 + 0.09296602×F3 (3-4)

**Supplementary Table 9.** Taxonomic alpha diversity (Shannon diversity and Chao richness) of microbial communities harboring nitrification related (*hao*) and denitrification related (*nirK*, *nirS*, *norB*, *norC* and *nosZ*) functional genes during vermicomposting.

| Compost samples | Nitrifier | |  | Denitrifiers | |
| --- | --- | --- | --- | --- | --- |
|  | Shannon | Chao |  | Shannon | Chao |
| T_0 | 0 | 0 |  | 1.94 | 110 |
| T_11 | 0 | 0 |  | 4.78 | 393 |
| T1_15 | 0.6 | 2 |  | 5 | 415 |
| T2_15 | 0.97 | 3 |  | 5.01 | 554 |
| T3_15 | 0 | 0 |  | 4.81 | 563 |
| T4_15 | 0.83 | 3 |  | 4.81 | 558 |
| T5_15 | 0.83 | 3 |  | 4.69 | 561 |
| T1_44 | 0 | 1 |  | 5.15 | 443 |
| T2_44 | 0 | 1 |  | 5.08 | 552 |
| T3_44 | 0 | 1 |  | 5.26 | 605 |
| T4_44 | 0.38 | 2 |  | 4.38 | 501 |
| T5_44 | 0 | 1 |  | 5.11 | 538 |
